# Hepatocellular Carcinoma Displays Distinct DNA Methylation Signatures with Potential as Clinical Predictors

**DOI:** 10.1371/journal.pone.0009749

**Published:** 2010-03-17

**Authors:** Hector Hernandez-Vargas, Marie-Pierre Lambert, Florence Le Calvez-Kelm, Géraldine Gouysse, Sandrine McKay-Chopin, Sean V. Tavtigian, Jean-Yves Scoazec, Zdenko Herceg

**Affiliations:** 1 Epigenetics Group, International Agency for Research on Cancer (IARC), Lyon, France; 2 Genetic Cancer Susceptibility Group, International Agency for Research on Cancer (IARC), Lyon, France; 3 Service d'Anatomie Pathologique, Edouard Herriot Hospital Lyon, Lyon, France; CNRS, France

## Abstract

**Background:**

Hepatocellular carcinoma (HCC) is characterized by late detection and fast progression, and it is believed that epigenetic disruption may be the cause of its molecular and clinicopathological heterogeneity. A better understanding of the global deregulation of methylation states and how they correlate with disease progression will aid in the design of strategies for earlier detection and better therapeutic decisions.

**Methods and Findings:**

We characterized the changes in promoter methylation in a series of 30 HCC tumors and their respective surrounding tissue and identified methylation signatures associated with major risk factors and clinical correlates. A wide panel of cancer-related gene promoters was analyzed using Illumina bead array technology, and CpG sites were then selected according to their ability to classify clinicopathological parameters. An independent series of HCC tumors and matched surrounding tissue was used for validation of the signatures. We were able to develop and validate a signature of methylation in HCC. This signature distinguished HCC from surrounding tissue and from other tumor types, and was independent of risk factors. However, aberrant methylation of an independent subset of promoters was associated with tumor progression and etiological risk factors (HBV or HCV infection and alcohol consumption). Interestingly, distinct methylation of an independent panel of gene promoters was strongly correlated with survival after cancer therapy.

**Conclusion:**

Our study shows that HCC tumors exhibit specific DNA methylation signatures associated with major risk factors and tumor progression stage, with potential clinical applications in diagnosis and prognosis.

## Introduction

Hepatocellular carcinoma (HCC) represents an endemic burden worldwide, partially due to delayed diagnosis and multiple risk factors that contribute to a permanent high incidence [1], [2]. Well-known risk factors include chronic hepatitis B virus (HBV) and hepatitis C virus (HCV) infection, toxic, metabolic and immune-related conditions [3]. In all these conditions, the development of malignancy is the consequence of a multistep process, including several morphologically recognizable stages and usually associated with a context of cirrhosis, a precancerous condition combining increased proliferation and prolonged environmental stress. The sequential progression to carcinoma has been related with changes at the genetic and epigenetic level [4]. A number of previous studies investigated genetic changes in HCC, including mutations and deletions in candidate cancer-associated genes [4]. Somatic mutations in several tumor suppressor genes (such as TP53, p16, and RB), oncogenes (including c-MYC and β-catenin), and other cancer-associated genes (including E-cadherin and cyclin D1) have been observed in HCC. These changes have been detected mainly in late stages of HCC development [4]. In addition, a frequent identification of loss of heterozygosity (LOH) in chromosome 8p in HCC cases, suggested that inactivation of the Deleted in Liver Cancer 1 gene (DLC-1) may play pivotal roles in HCC development [5]. However, while genetic events are likely to contribute to the development of HCC, neither of these genetic alterations has been consistently identified in HCC, suggesting that epigenetic changes may play an important role.

Aberrant DNA methylation is a major epigenetic mechanism of gene silencing and is observed in many human cancers [6], [7]. DNA methylation occurs in eukaryote DNA at CpG sites, usually enriched in the promoters of genes. In several types of tumors, including HCC, global hypomethylation and specific promoter hypermethylation have been linked with genomic instability and inactivation of tumor suppressor genes (TSG), respectively [8], [9]. Indeed, accumulating evidence indicates that HBV-infected hepatocytes often exhibit altered epigenetic status [10], [11]. In this sense, a deregulated methylation profile can be an early marker of disease and a useful tool for cancer screening. Several studies support the potential role of promoter hypermethylation in HCC-related gene silencing, and this has been shown to be positively correlated with tumor progression [12]. Relevant TSGs consistently found hypermethylated in HCC include *RASSF1A* or *p16INK4a* [12], [13], [14], [15], [16], [17], [18]. However, although a growing number of genes undergoing aberrant CpG island hypermethylation in HCC has been described, most studies have involved the analysis of hypermethylation in a limited number of gene promoters or a restricted number of HCC samples [12], [13], [14], [15], [16], [17], [18]. In addition to improving our understanding of liver carcinogenesis, large scale DNA promoter methylation profiles may produce useful associations with clinical parameters such as recurrence and survival.

We studied a series of human HCC samples for DNA promoter methylation using Illumina bead array analysis of 1505 CpG sites in 807 cancer-related gene promoters. Signatures of a distinct HCC methylation profile were obtained and validated, as well as their potential application as clinical predictors.

## Methods

### Patients and Biopsy Specimens

All patients included in the study were referred for treatment to Edouard Herriot Hospital in Lyon, France between 1997 and 2009. Tissue samples were used only from patients having signed an informed-consent form; all tumor tissue samples were obtained through the Tumorothèque des Hospices Civils de Lyon. The study was approved by the institutional review boards of the International Agency for Research on Cancer and the local ethics committee of Edouard Herriot Hospital.

38 patients with HCC were selected for analysis; in all cases, cryopreserved samples from the primary tumor were available for study; in 30 patients, paired cryopreserved samples of adjacent non malignant tissue were also available (for clinicopathological features, see Table 1). Samples from two patients with liver adenoma were used for comparison purposes. An additional series of 8 matched HCC and surrounding tissues was used for validation. In addition, three different human HCC cell lines (PLC/PRF/5, Hep3B, HepG2) and one breast carcinoma cell line (MCF7) were included in the array.

**Table 1:** Clinicopathological features of HCC patients.

Variable		No. of cases
No. of patients		30*
	Male	24
	Female	6
Age, mean ± SD		59±12.3
Etiology		
	HBV	9
	HCV	5
	Alcohol use	8
	Unknown risk factor	8
Tumor differentiation		
	Well	15
	Moderately	11
	Poorly	4
Tumor size		
	<5 cm	14
	>5 cm	16
TNM Stage		
	TI	14
	TII	6
	TIII	10
No. of nodules		
	Unilocular	14
	Multilocular	16
Cirrhosis		
	Yes	16
	No	14

* Only patients with paired samples (tumor and surrounding tissue) are described here.

For all patients, samples were taken from a surgical specimen, obtained through hepatectomy or liver transplantation, under the supervision of a pathologist; they were snap frozen less than 30 minutes after the removal of the surgical specimen and stored in liquid nitrogen until use. Before molecular analysis, the representativity and the quality of the sample were verified by a pathologist (Figure S1).

Information about risk factors for HCC was retrieved from clinical charts; the following information was noted: serological evidence for HBV or HCV infection, alcohol consumption, evidence for dysmetabolic syndrome or auto-immune disease, and other etiologies. Information about the evolution (treatments, duration of follow-up, duration of survival, status at the date of last information) was retrieved from clinical charts. The histological diagnosis and classification of primary liver tumors and the histological evaluation of the adjacent liver tissue were performed by an experienced pathologist (JYS).

### Bead array analysis of DNA promoter methylation

Tissues were frozen in liquid nitrogen, ground into powder and then collected into eppendorf tubes. Genomic DNA from HCC tumors and surrounding tissue was prepared by overnight proteinase K treatment, phenol-chloroform extraction, and ethanol precipitation. Sodium bisulfite modification was performed on 500 ng DNA using the EZ DNA Methylation-Gold Kit (Zymo Research). DNA methylation profiling using bead arrays for 1505 CpG sites, corresponding to 807 cancer-related genes, was performed with the Illumina GoldenGate methylation assay (Illumina) as described previously [19]. Briefly, for each CpG site, four probes are included: two allele-specific oligos (ASO) and two locus-specific oligos (LSO). Each ASO–LSO oligo pair corresponds to either the methylated or unmethylated state of the CpG site. Each methylation data point is represented by two-color fluorescent signals from the M (methylated) and U (unmethylated) alleles. Technical replicates of several bisulfite-converted samples were run. BeadStudio v3.2 software (Illumina) was used for initial filtering and clustering analysis (see below).

### Pyrosequencing

Genomic DNA from HCC tumors and surrounding tissue was extracted and modified as described above. The eluted DNA was at a final concentration of 25 ng/µl. To quantify the percentage of methylated cytosine in individual CpG sites, bisulfite-converted DNA was sequenced using a pyrosequencing system (PSQ™ 96MA, Biotage, Sweden) [20]. This method treats each individual CpG site as a C/T polymorphism and generates quantitative data for the relative proportion of the methylated versus the unmethylated allele. Pyrosequencing assays were established for the quantitative measurement of DNA methylation levels in the promoter region of 8 genes (*RASSF1, GSTP1, APC, GNMT, GABRA5*, *MEST*, *MGMT*, and *H19*), and LINE-1 using primers previously described [21]. (*Table S1* and *Figure S2*). Hot-start PCR was performed with HotStarTaq Master Mix kit (Qiagen), and pyrosequencing was carried out in accordance with the manufacturer's protocol (Biotage). The target CpGs were evaluated by converting the resulting pyrograms into numerical values for peak heights, and calculating the average of all CpG sites analyzed at a given gene promoter (Figure S2).

### Quantitative RT-PCR

Total RNA was isolated using the TRIzol Reagent (Invitrogen) according to the manufacturer's instructions. Reverse transcription reactions were performed using MMLV-RT (Invitrogen) and random hexamers, according to the manufacturer's protocol. Primers and probes were designed using Universal Probe Library Assay Design Center (Roche). Quantitative real-time PCR (qRT-PCR) was performed in triplicates of each condition, using FastStart TaqMan Probe Master (Roche) and a MXP3000 real-time PCR system (Stratagene).

### Statistical Analysis

#### Filtering and unsupervised clustering

BeadStudio version 3.2 (Illumina) was used for obtaining the signal values (AVG-Beta) corresponding to the ratio of the fluorescent signal from the methylated allele (Cy5) to the sum of the fluorescents signals of both methylated (Cy5) and unmethylated alleles (Cy3), 0 corresponding to completely unmethylated sites and 1 to completely methylated sites. In order to avoid the gender effect, all probes in chromosome X (n = 84) were discarded. In addition, all probes with a *P* value above 0.01 in more than 10% of the samples were excluded from the analysis. BRBArrayTools software (version 3.8 beta2) was used for further analysis, using the AVG-Beta values. CpG sites showing minimal variation across the set of arrays were excluded from the analysis. Gene ontology and molecular interactions were analyzed with GenMAPP version 2.1 (http://GenMAPP.org/), and the KEGG Pathways Database (http://www.genome.jp/kegg/). Unsupervised hierarchical clustering, class comparison, class prediction, KEGG pathway enrichment, and survival prediction were performed with the BRBArrayTools software.

#### Class Comparison

CpG sites were considered differentially methylated when their *P* value was less than 0.001. In addition, we identified CpG sites that were differentially methylated between tumor and adjacent tissue by using a multivariate permutation test [22] providing 90% confidence that the false discovery rate was less than 10%. The false discovery rate is the proportion of the list of CpG sites claimed to be differentially methylated that are false positives. The test statistics used are random variance t-statistics for each CpG site [23]. Although t-statistics were used, the multivariate permutation test is non-parametric and does not require the assumption of Gaussian distributions. A global test of whether the methylation profiles differed between the classes was also performed by permuting the labels of which CpG methylation states corresponded to which classes. For each permutation, the *P* values were re-computed, and the number of CpG sites significant at the 0.001 level was noted. The proportion of the permutations that gave at least as many significant CpG sites as with the actual data was the significance level of the global test (*P*<0.05 for the global test).

In addition, we performed an alternative analysis considering the frequency of methylation in tumors respective to surrounding tissue. To this end, we defined a threshold for frequently unmethylated and frequently methylated genes based on the 25 and 75 percentiles in the surrounding tissues, respectively. This is, a given CpG site was considered as frequently hypermethylated in tumors if more than 75% of the tumor samples lied above the 75 percentile in surrounding tissues. Similarly, if more than 75% of the tumor samples lied below the 25% of methylation in surrounding samples, this CpG site was considered as frequently hypomethylated in tumors (Figure S3).

#### Class Prediction

We used different models to predict the class of future samples using CpG methylation profile based on the Compound Covariate Predictor [24], Diagonal Linear Discriminant Analysis [25], Nearest Neighbor Classification [25], and Support Vector Machines with linear kernel [26]. The models incorporated CpG sites that were differentially methylated at the 0.001 significance level as assessed by the random variance t-test [23]. We estimated the prediction error of each model using leave-one-out cross-validation (LOOCV) [27]. For each LOOCV training set, the entire model building process was repeated, including the gene selection process. We also evaluated whether the cross-validated error rate estimate for a model was significantly less than one would expect from random prediction. The class labels were randomly permuted and the entire LOOCV process was repeated. The significance level is the proportion of the random permutations that gave a cross-validated error rate no greater than the cross-validated error rate obtained with the real data. 1000 random permutations were used.

In addition, the Prediction Analysis for Microarrays (PAM) Tool was used as another method of class prediction. The method uses the shrunken centroid algorithm [28], whereby the centroids of each group are shrunken toward each other by shrinking the class means of each CpG site toward an overall mean. The amount of shrinking is determined by a “tuning parameter” called delta. As the shrinking occurs, some CpG sites will have the same value of shrunken class mean for the different classes, and hence they will have no effect in distinguishing the classes. For larger values of delta, fewer CpG sites will have different shrunken means among the classes, and so the classifier will be based on fewer CpG sites. With this approach, the number of CpG sites included in the classifier is determined by the value of delta. The algorithm provides a k-fold cross-validated estimate of prediction error for all values of delta where k is the minimum class size. The tool indicates the delta corresponding to the smallest cross-validated prediction error and gives the list of CpG sites that are included in the classifier for that value of delta.

#### Gene Ontology Analysis

The evaluation of which Gene Ontology (GO) classes are differentially methylated between tumor and surrounding samples was performed using a functional class scoring analysis as previously described [29]. For each gene in a GO class, the *P* value for comparing tumor and surrounding samples was computed. The set of *P* values for a class was summarized by two summary statistics: (i) The LS summary is the average log *P* values for the genes in that class and (ii) the KS summary is the Kolmogorov-Smirnov statistic computed on the *P* values for the genes in that class. Functional class scoring is a more powerful method of identifying differentially methylated gene classes than the more common over-representation analysis or annotation of gene lists based on individually analyzed genes. The functional class scoring analysis for GO classes was performed using BRB-ArrayTools.

#### Survival Analysis

CpG sites whose methylation was significantly related to overall survival after treatment were selected with BRB-ArrayTools survival analysis. A statistical significant level was computed for each gene based on univariate proportional hazards models. These *P* values were then used in a multivariate permutation test in which the survival times and censoring indicators were randomly permuted among arrays [27], [30]. The multivariate permutation test was used to provide 90% confidence that the false discovery rate was less than 10%.

For other comparisons, means and differences of the means with 95% confidence intervals were obtained using GraphPad Prism (GraphPad Software Inc.). The Mann-Whitney test and the Wilcoxon matched pairs test were used for unpaired and paired analysis comparing average methylation between classes, respectively. *P* values<0.05 were considered statistically significant.

## Results

### DNA promoter methylation in HCC samples

**Figure 1:**
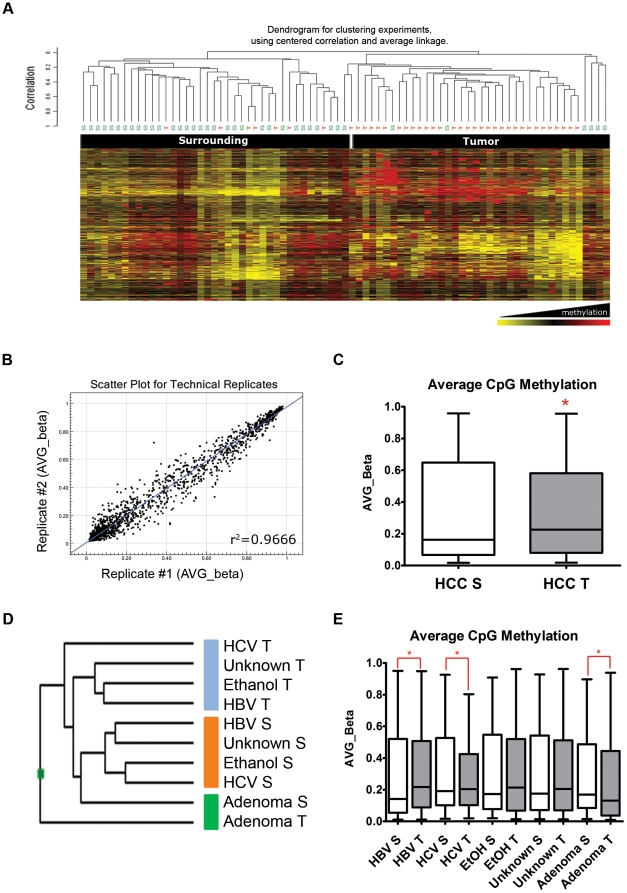
Unsupervised analysis of CpG methylation bead arrays in HCC. **A.** Clustering analysis of 76 HCC samples included in the bead array assay (HCC tumor and surrounding tissue). For the upper part of the cluster, names are given manually according to the enrichment of specific clusters. 1505 CpG sites are included. Yellow indicates hypomethylated, and red hypermethylated CpG sites. **B.** Representative logarithmic plot of two replicates included in the array, showing proper consistency of methylation (r2 value is included on the plot). **C.** Average promoter methylation of all 1505 CpG sites, in HCCs and surrounding tissues. **D.** Clustering analysis after grouping the samples by ethological factors. **E.** Average methylation for all 1505 CpG sites from the same ethological groups shown in (d). Significant differences (P<0.05) between tumor and surrounding tissue are represented with an asterisk (*).

To investigate whether HCC could harbor specific methylation profiles, DNA methylation of 1505 CpG sites was analyzed using Illumina bead arrays. A total of 38 HCC samples were suitable for analysis, including 30 pairs of HCC tumors/surrounding tissues. In addition, 4 liver adenoma tumors/surrounding samples and 4 cancer cell lines were included for comparison. 1219 Probes were used in the analysis, after excluding those with a *P* value higher than 0.01 in more than 10% of the samples, and those in chromosome X (to avoid the gender effect). An initial unsupervised hierarchical clustering analysis was able to distinguish HCC samples from other types of tumors (breast and esophageal cancer), blood and cell lines (data not shown). Unsupervised clustering within HCC samples was also able to distinguish 2 clusters enriched in tumors and surrounding tissue samples (Figure 1A). Together with the proper clustering of the replicates in the unsupervised analysis, the scatter plots analysis confirmed the quality and reproducibility of the methylation profiling (Figure 1B).

Overall, tumor samples displayed a small but significant increase in average promoter CpG methylation (median methylation of 0.16 and 0.23 for surrounding and tumor tissue, respectively, *P*<0.05) (Figure 1C). This contrasts with the global DNA methylation as assessed with the LINE-1 element [21], which shows a significant hypomethylation in tumors compared to surrounding tissue (*P*<0.005, Figure S2C). An unsupervised analysis of samples grouped by risk factors (HBV, HCV, alcohol consumption, or unknown risk) showed that surrounding tissues were clustered together, while tumor tissues were in a separate group among which HCV-associated HCC were the most divergent subset (Figure 1D). When analyzing the average promoter methylation for these groups, an increased methylation was consistently found in tumor samples relative to surrounding tissue, with the exception of adenoma samples (Figure 1E). This increase in average promoter methylation was statistically significant for HBV and HCV samples (*P*<0.0001 for both paired analysis). Although promoter methylation was also increased in alcohol-related and unknown-risk HCC samples, the difference did not reach statistical significance. Therefore, a distinct promoter methylation profile is common to all HCC tumors, with global non-promoter hypomethylation and increased promoter methylation.

### Signature and prediction of HCC by DNA promoter methylation profiling

**Figure 2:**
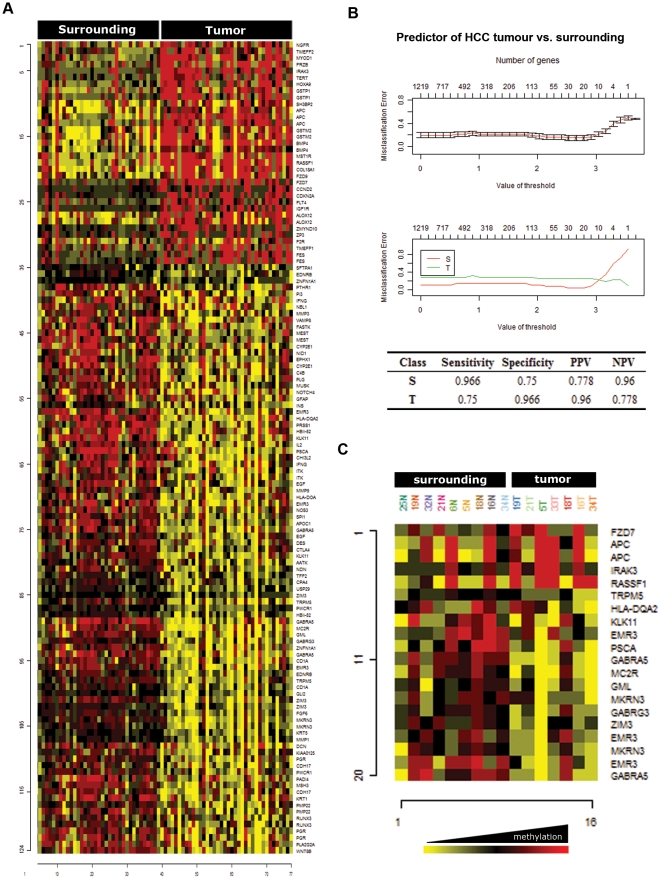
Signature and predictor of HCC by methylation profiling. **A.** Differential methylation analysis was performed with the class comparison tool of BRBArrayTools software, as described in Materials and Methods. The heat map represents those CpG sites distinguishing HCC from surrounding tissue (n = 87) with a P value<0.001. The full list of CpG sites is presented as Table S2. Yellow indicates hypomethylated, and red hypermethylated CpG sites. **B.** Representation of the misclassification error as a function of the number of genes, as assessed with the PAM prediction analysis. The upper panel shows the correlation for the grouped samples; the lower panel shows the independent correlation for tumor and surrounding samples. Sensitivity and specificity of the predictor is included in the Figure. **C.** A heat map with the 20 CpG sites included in the HCC predictor was obtained for an independent series of HCC samples and HCC surrounding tissues, after unsupervised hierarchical clustering analysis.

To distinguish those genes differentially methylated between tumors and surrounding tissue, a class comparison tool (BRBArrayTools v3.8) was used, as described in *Methods*. After filtering for a *P* value<0.001 and correcting for a False Discovery Rate (FDR) <0.1, 124 CpG sites were shown to be differentially methylated. Several CpG sites corresponded to the same gene promoter, and consequently a total of 94 genes were considered as differentially methylated. Approximately one third of the significant promoters were significantly represented by more than one CpG site, arguing in favor of the quality of this data. Relative to surrounding tissues, tumors showed increased methylation in 34 (27%) of these CpG sites (corresponding to 27 gene promoters, including *RASSF1*, *APC*, and *CDKN2A*), and reduced methylation in 90 (73%) (corresponding to 66 gene promoters, including *GABRA5*, *NOTCH4*, and *PGR*) (Figure 2A and Table S2). To analyze the frequency of methylated or unmethylated CpG sites in tumors relative to surrounding tissue we used the upper and lower quartile of surrounding tissue to set a threshold (see Methods). This analysis yielded a similar result, with 7 and 35 CpG sites respectively hyper- and hypomethylated in tumors (Figure S3).

Validation of a subset of 8 gene promoters by pyrosequencing was consistent with the bead arrays results (Figure S4A). The correlation between pyrosequencing and bead array analysis was statistically significant (*P* value<0.0001, Figure S4B). In addition, hypermethylation of *RASSF1A* and of *APC* promoters was associated with a significantly lower expression in HCC tumors, as assessed by qRT-PCR (Figure S5).

The ontological analysis of the differentially methylated genes showed enrichment for ontology terms related to development, including the Wnt-β−catenin, TGF-β, Hedgehog and Notch signaling pathways (data not shown). Methylation of some of these genes has been previously described in HCC (i.e. *APC*, *RASSF1A*, and *p16/CDKN2A*), validating the sensitivity of this assay [14], [31], [32]. However, many gene promoters that were not previously linked to HCC showed differential methylation, including those involved in apoptosis (*IRAK3*, *MYOD1*), immune response (*HLA-DQA2*, *GSTM2*, *IFNG*), growth factor signaling (*EGF*, *FGF6*, *IGF1R*, *NGFR*), cell cycle regulation (*CCND2*), and metastasis (*CDH17*, *MMP1*, *MMP3*, *MMP9*) (Table S2). Interestingly, promoters in the HCC signature included a number of imprinted genes that were consistently hypomethylated in HCC relative to surrounding tissue (*GABRA5*, *GABRG3*, *HBII-52*, *MEST*, *MKRN3*, *TRPM5*, and *ZIM3*). For most of them there were at least 2 CpG sites differentially methylated, suggesting that this observation is biologically significant.

The ability to discriminate tumor from surrounding tissue may have clinical impact, especially when small sets of genes are able to produce robust predictions. The significant differences between surrounding and HCC tissues after class comparison suggested the possibility of building a multivariate predictor from this gene set. Therefore, we next used a subset of CpG sites to predict the class of an independent series of HCC tumors and matching surrounding tissues. The models incorporated genes that were differentially methylated between tumor and surrounding tissue at the 0.001 significance level, as assessed by the random variance t-test. The prediction error of each model was assessed using leave-one-out cross-validation (LOOCV) [27]. Interestingly, the 124 CpG sites included in the HCC signature were able to discriminate tumor and surrounding tissue in all the samples included in the second series (data not shown).

We next tried to design a predictor with a minimum number of CpG sites using the Prediction Analysis of Microarrays tool (PAM) [28]. As shown in Figure 2B, a minimum of 20 CpG sites is required to minimize the number of misclassification errors. This 20 CpG site predictor (corresponding to 16 gene promoters) was able to correctly classify 14 out of 16 of the new samples (sensitivity = 0.75, specificity = 0.97 for tumor prediction), and was included in the 124 CpG sites signature of HCC. An unsupervised clustering for the new series of HCC samples using this 20 CpG sites-signature highlights its ability to discriminate both types of samples (Figure 2C). Interestingly, the CpG sites with strongest ability to discriminate tumor from surrounding tissue were found in the promoter of genes hypermethylated in HCC samples (e.g. *APC*, *RASSF1A*, *CDKN2A*, and *FZD7*).

### Methylation profile is associated with HCC risk factor and tumor progression

**Figure 3:**
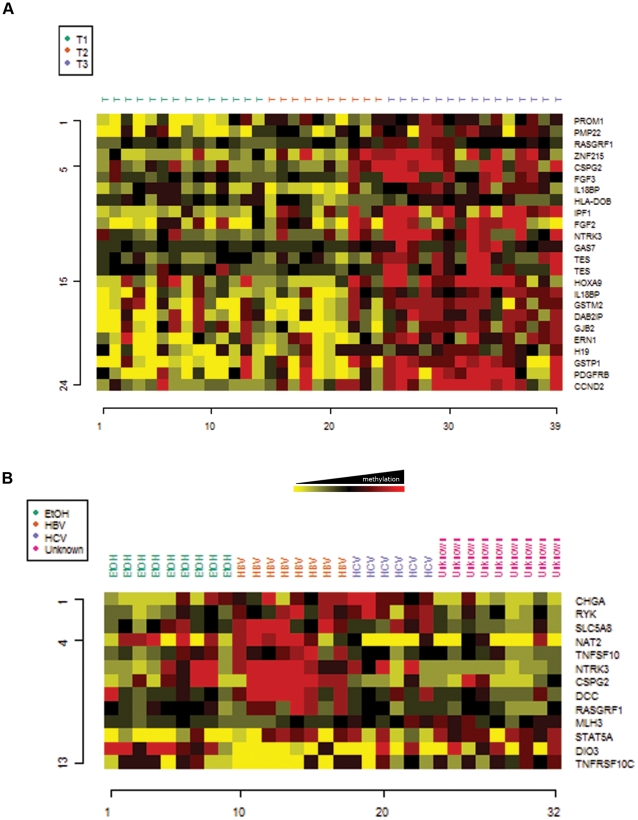
Methylation profile according to risk factor and tumor progression. Class comparison analyses were performed, as described in Figure 2. **A.** The heat map represents 27 CpG sites distinguishing the different HCC samples according to their TNM classification, with a P value<0.05. **B.** The heat map represents 17 CpG sites distinguishing the different HCC samples according to their ethological exposure, with a P value<0.01. HBV or HCV infection, EtOH  =  ethanol consumption, and Unknown  =  unknown risk factor.

In order to find CpG sites potentially associated with tumor progression, we performed a class comparison analysis to classify the methylation profile according to tumor stage (as assigned by the TNM classification) and grade of differentiation (histologically classified as 1  =  well differentiated, 2 =  intermediate, and 3 =  poorly differentiated). Tumor stage will be referred to as T, as all samples except one [T3N1M0] sample were negative for lymph node invasion (N0) and metastasis (M0). Globally, tumors of the first 2 stages (T1 and T2) displayed a similar methylome profile, while 24 CpG sites were differentially methylated in advanced tumors (T3) (Figure 3A). All CpG sites were significantly hypermethylated in advanced tumors, and most of them show a trend to be progressively hypermethylated from T1 through T3 (Figure 3A). The set of 24 CpG sites hypermethylated in advanced HCC tumors are located in genes involved in immune response and adhesion (*IL18BP*, *IPF1*, *HLA-DOB*, *CSPG2*, *GJB2* and *PMP22*), and the cell cycle (*CCND2* and *NTKR3*). Similarly, the grade of differentiation was associated with changes in methylation only in the least differentiated tumors (grade 3) (data not shown). Three CpG sites were significantly hypomethylated in grade 3 tumors (e.g. *HOXB2*, *DDR2*, and *TIMP3*), while 19 CpG sites were hypermethylated (including *CDK2*, *EF3*, *FANCF*, *LIF*, *RASGRF1*, *DNMT1*, and *ERCC1*).

The HCC samples analyzed in this study were obtained from patients exposed to different risk factors, including HBV infection, HCV infection, and ethanol consumption. In order to identify risk factor-specific profiles of methylation we performed a class comparison analysis including these groups, and a group of HCC samples with unknown risk factors (negative for HBV or HCV infection, and no history of alcohol consumption). After class comparison analysis, a reduced set of genes was significantly hypermethylated in each group relative to the other 3 groups (Figure 3B). By comparing among these groups it was possible to select CpG sites specifically modulated in alcohol-related (*DIO3* and *STAT5A*), HBV-related (*NAT2*, *CSPG2*, *DCC*, *NTKR3*, *TNFSF10*, *TNFRSF10C*, and *RASGRF1*), and HCV-related HCCs (*RIK* and *CHGA*). Samples from unknown risk factor patients displayed a mixed profile, with hypermethylation of several of these promoters, probably reflecting their heterogeneous origin (Figure 3 and Table S3).

The heterogeneity of HCC origin is also reflected in the conservation of the normal architecture of the liver. In this sense, our series of HCC surrounding tissues can be classified into those samples exhibiting cirrhotic (n = 16) or non-cirrhotic (n = 14) histology. Comparison between these two classes using stringent conditions of analysis (*P* value<0.001) shows that cirrhotic tissues are significantly hypermethylated in 2 gene promoters, corresponding to *UGT1A7* and *PLG*.

### HCC methylation profile and prediction of survival

**Figure 4:**
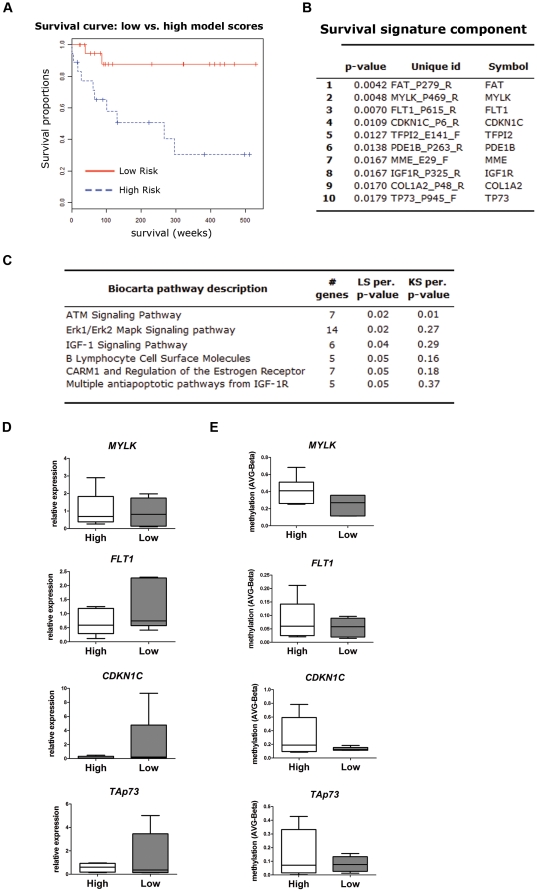
Survival risk predictor in HCC. **A.** Survival analysis using BRB-ArrayTools. A survival signature was developed using fitted Cox proportional-hazards model and leave-one-out crossvalidation, considering the time of biopsy as the starting point. Survival curves show a significant difference between two groups of HCC patients. **B.** A 58 CpG sites predictor (selected from the analysis shown in **A.**) was correlated with survival after treatment. Only the first 10 CpG sites (with the lowest P value) are shown. **C.** Pathway analysis for the 58 CpG sites included in the survival predictor showing the 5 significantly enriched pathways. **D.** Quantitative RT-PCR was performed for some of the genes with the highest ability to predict survival in HCC (MYLK, FLT1, CDKN1C and TAp73, in a subset of samples with high (H) and low (L) risk.

Survival signatures were developed with BRB-ArrayTools using fitted Cox proportional hazards model, considering the time of biopsy as the starting point. At the time of analysis there were 13 deaths among 38 patients with available data, with a mean follow-up time of 194 weeks for all patients. With these data it was possible to classify the patients into two groups with significantly different survival curves (Figure 4A, *P*<0.001). The first 10 CpG sites with highest ability to differentiate between these two groups are shown in Figure 4B. Interestingly, this survival signature was significantly enriched in the promoters of genes involved in IGF-1 signaling and immune response (Figure 4C). In addition, the differences found in DNA promoter methylation were reflected in different expression profiles for some of the genes ranking highest in the survival prediction analysis (Figure 4D). This suggests that control of immune and growth factor response genes by methylation may represent a potential mechanism directly affecting the survival of HCC patients.

## Discussion

This report describes the CpG methylation profile of HCC in a wide panel of cancer-related promoters. A differential analysis identified a signature of the genes specifically methylated in HCC with respect to surrounding tissue. Although a number of known promoters were found to be differentially methylated in HCC, we identified new candidate promoters that are potentially involved in the development and progression of liver cancer. By correlating the methylation data with clinical outcomes it was possible to establish a DNA methylation predictor of patient survival and clinical parameters such as stage and grade. The strength and low complexity of these signatures, based on a reduced number of gene promoters, makes them a potential novel strategy for early detection and clinical prediction in HCC.

Although early detection of HCC has improved, diagnosis is established at only advanced stages. Therefore, there is an urgent need to predict recurrence and response to therapy, especially because patients prone to recurrence may receive alternative treatment. The strength of the presented signatures is underscored by their validation in an independent series of HCC samples. Importantly, despite preliminary studies on clinical prediction based on gene expression profiling [33], the stability of DNA relative to RNA makes methylation profiling a tool better suited to clinical settings. In addition, the availability of signatures with a reduced number of CpG sites would enable their use for clinical prediction in, for example, paraffin-embedded samples or plasma DNA. A small set multivariate predictor may have important applications in the early detection of neoplastic transformation in populations at high risk for HCC, such as hereditary haemochromatosis patients [18]. Similarly, the prediction of survival may be useful in improving and individualizing therapeutic decisions. However, these multivariate signatures should be prospectively validated in larger cohorts before considering clinical applications.

The importance of the role of DNA methylation has been previously described in HCC. Epigenetic changes on RASSF1A, p16, and p15 tumor suppressor genes in serum DNA have been shown to be potential biomarkers for early detection in populations at high risk for HCC [18]. The tumor suppressor APC also seems to be a common marker for HCC detection and is found consistently hypermethylated in HCC [12], whereas SYK and CRABP1 hypermethylation has been considered as a useful prognostic marker in HCC [34]. A previous screening of 105 promoters identified that the epigenetic activation of Ras and downstream Ras effectors was common in HCC, and was associated with poor prognosis [8]. In another study, increased methylation was shown in the p16 and GSTP1 genes in HCC compared to matching non-malignant cirrhotic liver [12], [35], [36]. In this sense, our bead array analysis supports and extends the previous findings on DNA methylation, and provides a novel and more comprehensive signature of HCC methylation.

A previous study analyzed a limited panel of cancer-associated genes in HCC tumors and found that environmental factors may influence the degree and pattern of methylation in tumors [37]. Our study identified significant associations between methylation patterns and specific etiologic agents (i.e., HBV, HCV, and ethanol), tumor progression (stage and grade of differentiation), and tumor background (cirrhotic vs. non-cirrhotic surrounding tissue) for specific subsets of genes. Interestingly, those promoters differentially methylated in virus-related HCC samples correspond to genes involved in immune response and induction of apoptosis. Specifically, polymorphisms of the N-acetyltransferase encoded by the *NAT2* gene have been linked to susceptibility to HBV-related HCC [38], [39]. Moreover, promoter methylation of *DNMT1* was associated with poor differentiation.. Remarkably, hypermethylation of the gene encoding DNA-methyltransferase 1 (DNMT1) can be associated with a lower expression and consequent global hypomethylation as observed with the LINE-1 pyrosequencing analysis.

Another interesting observation is that the tumor background (cirrhotic vs. non-cirrhotic) determined a specific pattern of methylation for several promoters. *UGT1A7* encodes a UDP-glucuronosyltransferase involved in multiple metabolic pathways, including the metabolism of hormones and the metabolism of xenobiotics by cytochrome P450. In addition, *UGT1A7* polymorphisms have been correlated with cirrhosis, and with increased risk of HCC in HBV- and HCV-infected patients [40], [41], [42]. Similarly plasminogen, encoded by *PLG*, is a circulating zymogen that is converted to the active enzyme plasmin and whose main function is to dissolve fibrin clots. It is noteworthy that *PLG* transcript expression has been reported to be reduced in HCC [43]. Therefore, aberrant promoter methylation of these two genes may be related with a disturbed detoxification of carcinogens, and the process of hepatic fibrogenesis that results in cirrhosis [44]. Further analysis of these genes may shed new light into the process of liver carcinogenesis in specific risk groups. However, the global similarity among HCC groups substantiates the notion that aberrant methylation is a ubiquitous phenomenon in liver carcinogenesis [8].

In summary, this study describes the methylation profile of hepatocellular carcinoma and the specific signatures that can be used as markers for detection and survival after therapy. Our results, based on bead arrays and quantitative analysis with pyrosequencing, give a reliable view of HCC promoter methylation in a wide panel of genes, and can be used as a reference tool for the potential development of clinical applications.

## Supporting Information

Figure S1 Representative histology of HCC tumors and surrounding tissues used for methylation profiling. H&E-stained HCC samples with surrounding non-tumor liver parenchyma. Examples of HCC samples with adjacent non-cirrhotic and cirrhotic tissues are shown in A and B, respectively. NC indicates non-cirrhotic surrounding liver tissue, C indicates cirrhotic surrounding liver tissue, and H indicates HCC tissue.
(7.59 MB TIF)

Figure S2 Pyrosequencing design for imprinted genes. A. Diagram showing chromosomal localization and GC percentage for GABRA5 promoter, as an example of the design used for validation. The regions studied by bead arrays and pyrosequencing are represented under the chromosomal localization. B. Representative pyrograms of GABRA5 obtained from the analysis of bisulfite-modified DNA from HCC tumor and surrounding tissue. Primers used for pyrosequencing are included as Supplementary Table 1. C. Global methylation was studied using primers against LINE-1 elements [21]. A significant hypomethylation in tumors, relative to surrounding tissue, is shown by a (*) (<0.05).
(1.54 MB TIF)

Figure S3 Analysis of frequency of methylation. AVG-Beta values in the surrounding tissues were used to define the percentiles 25 and 75 for each CpG site (see Methods). These percentiles were used as a reference to define the frequency of methylation in tumors. A. Box plots representing the 3 CpG sites with highest frequency of methylation in tumors (upper panel) and highest frequency of unmethylation in tumors (lower panel) calculated in this way. S  =  surrounding, T  =  tumor. (*) P value < 0.001. B. Table showing the CpG sites frequently methylated in more than 75% of the tumors relative to surrounding tissues. C. Table showing the CpG sites frequently unmethylated in more than 75% of the tumors relative to surrounding tissues.
(2.56 MB TIF)

Figure S4 Validation of bead arrays by pyrosequencing A. Pyrosequencing assays were designed for the validation of 8 gene promoters differentially methylated between tumor and surrounding HCC samples (upper dot plot). The level of methylation is shown in a percentage scale. Primers were designed as described in Materials and Methods. A dot plot representing the corresponding levels of methylation (in a 0 to 1 scale) for the same genes in the bead arrays assay is shown in the lower panel. B. Correlation analysis from the data presented in (A).
(1.45 MB TIF)

Figure S5 Validation of bead arrays by qRT-PCR Quantitative RT-PCR was performed for APC and RASSF1A in a subset of samples. The bars show a lower expression in the tumors relative to surrounding tissue in 3 out of 4 samples analyzed. In addition, inverse correlation with methylation is shown in each plot. Each line represents the AVG-Beta value obtained with bead arrays for 2 independent probes in the same promoter. Higher initial methylation is observed for the last sample, in which expression in tumors is higher than the matched surrounding tissue.
(2.17 MB TIF)

Table S1 Primers used for pyrosequencing.
(0.06 MB DOC)

Table S2 CpG sites differentially methylated in HCC tumor vs. surrounding tissue.
(0.25 MB DOC)

Table S3 CpG sites differentially methylated in HCC according to risk factor exposure.
(0.07 MB DOC)
