# Supplementary figures and images for: Hepatocellular Carcinoma Displays Distinct DNA Methylation Signatures with Potential as Clinical Predictors
Source: PLoS One. 2010 Mar 17;5(3):e9749. doi: 10.1371/journal.pone.0009749 (PMC2840036; doi:10.1371/journal.pone.0009749)

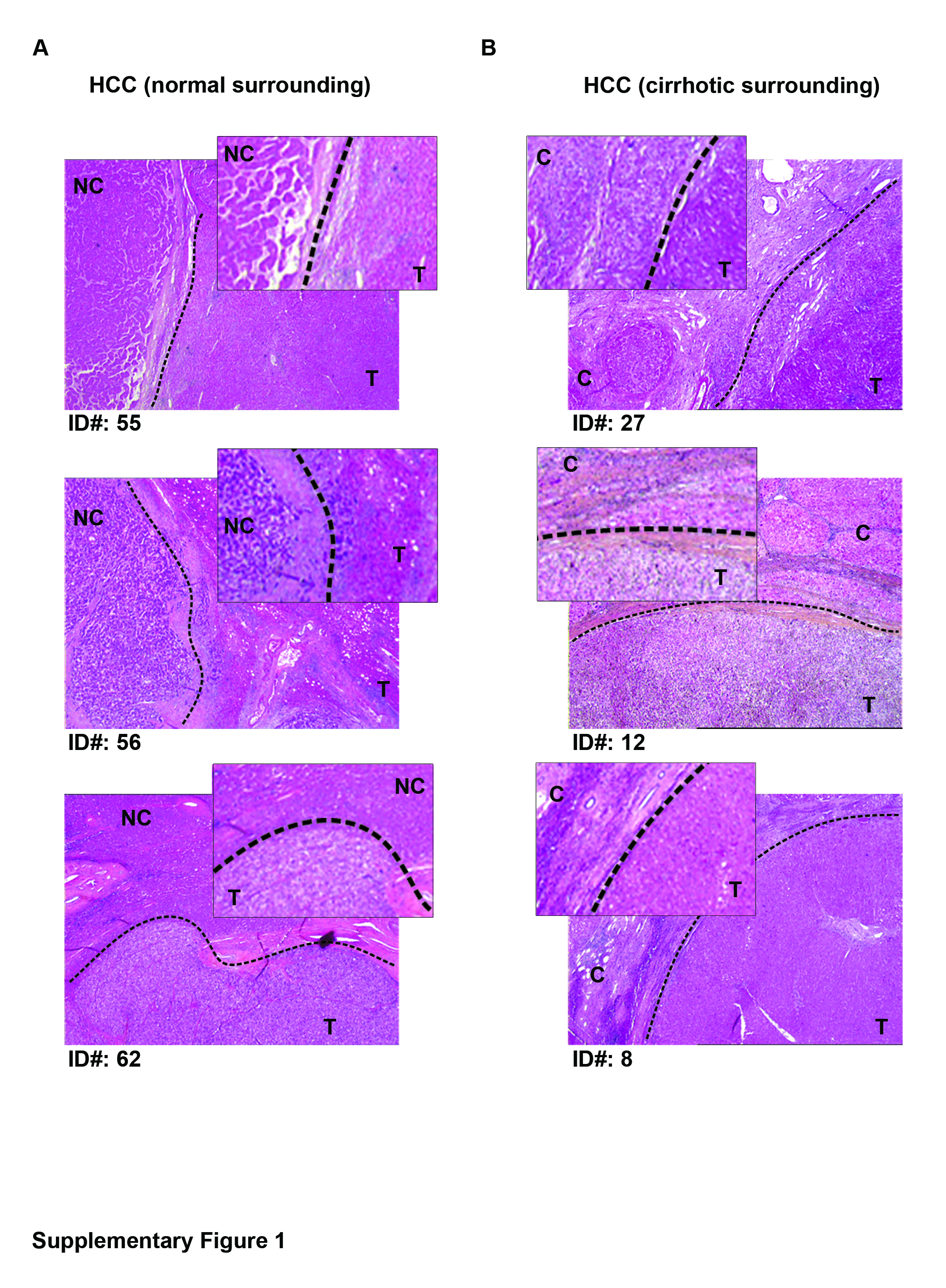

Supplement: Figure S1 — Representative histology of HCC tumors and surrounding tissues used for methylation profiling. H&E-stained HCC samples with surrounding non-tumor liver parenchyma. Examples of HCC samples with adjacent non-cirrhotic and cirrhotic tissues are shown in A and B, respectively. NC indicates non-cirrhotic surrounding liver tissue, C indicates cirrhotic surrounding liver tissue, and H indicates HCC tissue. (7.59 MB TIF) [file pone.0009749.s001.tif]

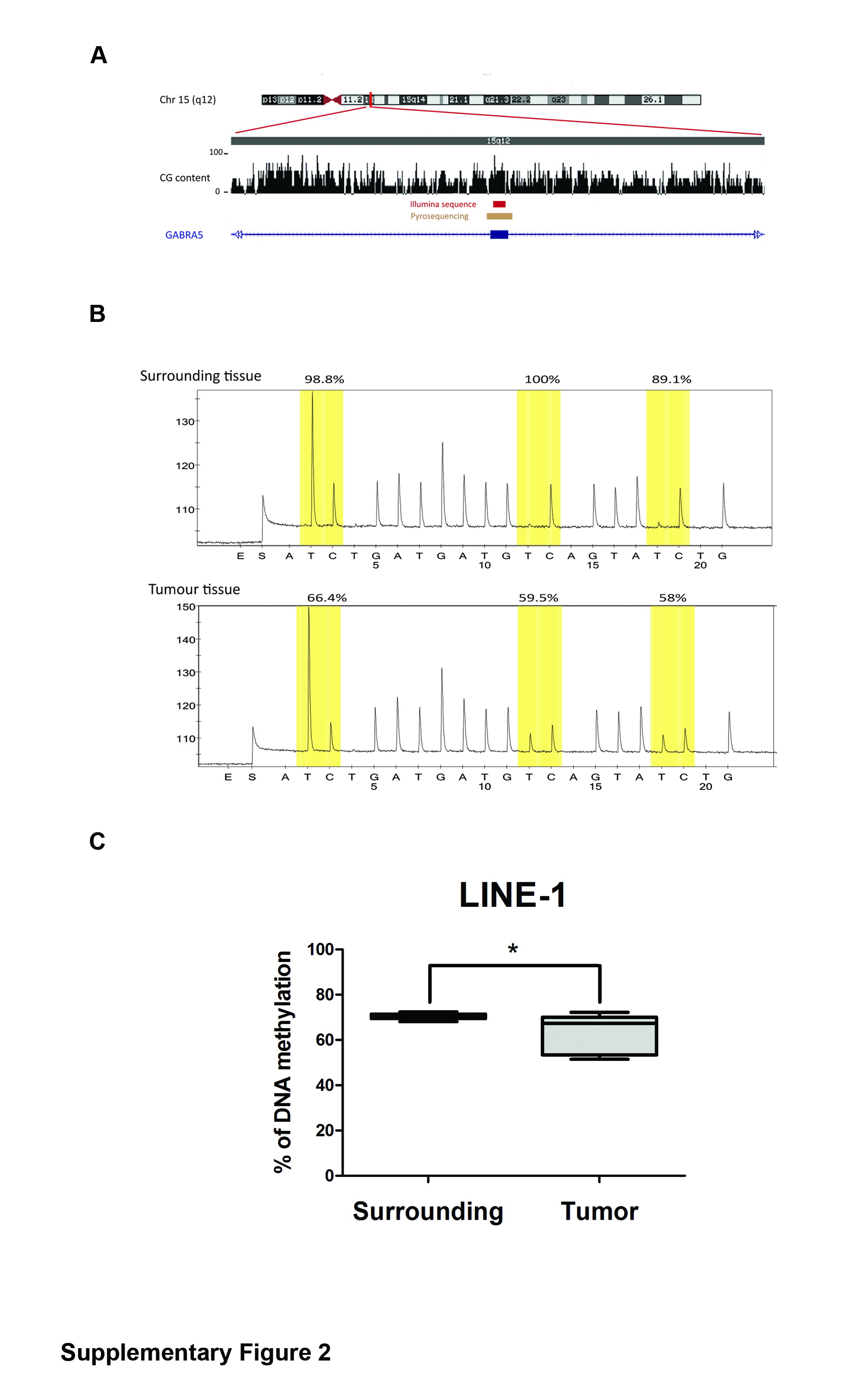

Supplement: Figure S2 — Pyrosequencing design for imprinted genes. A. Diagram showing chromosomal localization and GC percentage for GABRA5 promoter, as an example of the design used for validation. The regions studied by bead arrays and pyrosequencing are represented under the chromosomal localization. B. Representative pyrograms of GABRA5 obtained from the analysis of bisulfite-modified DNA from HCC tumor and surrounding tissue. Primers used for pyrosequencing are included as Supplementary Table 1. C. Global methylation was studied using primers against LINE-1 elements [21]. A significant hypomethylation in tumors, relative to surrounding tissue, is shown by a (*) (<0.05). (1.54 MB TIF) [file pone.0009749.s002.tif]

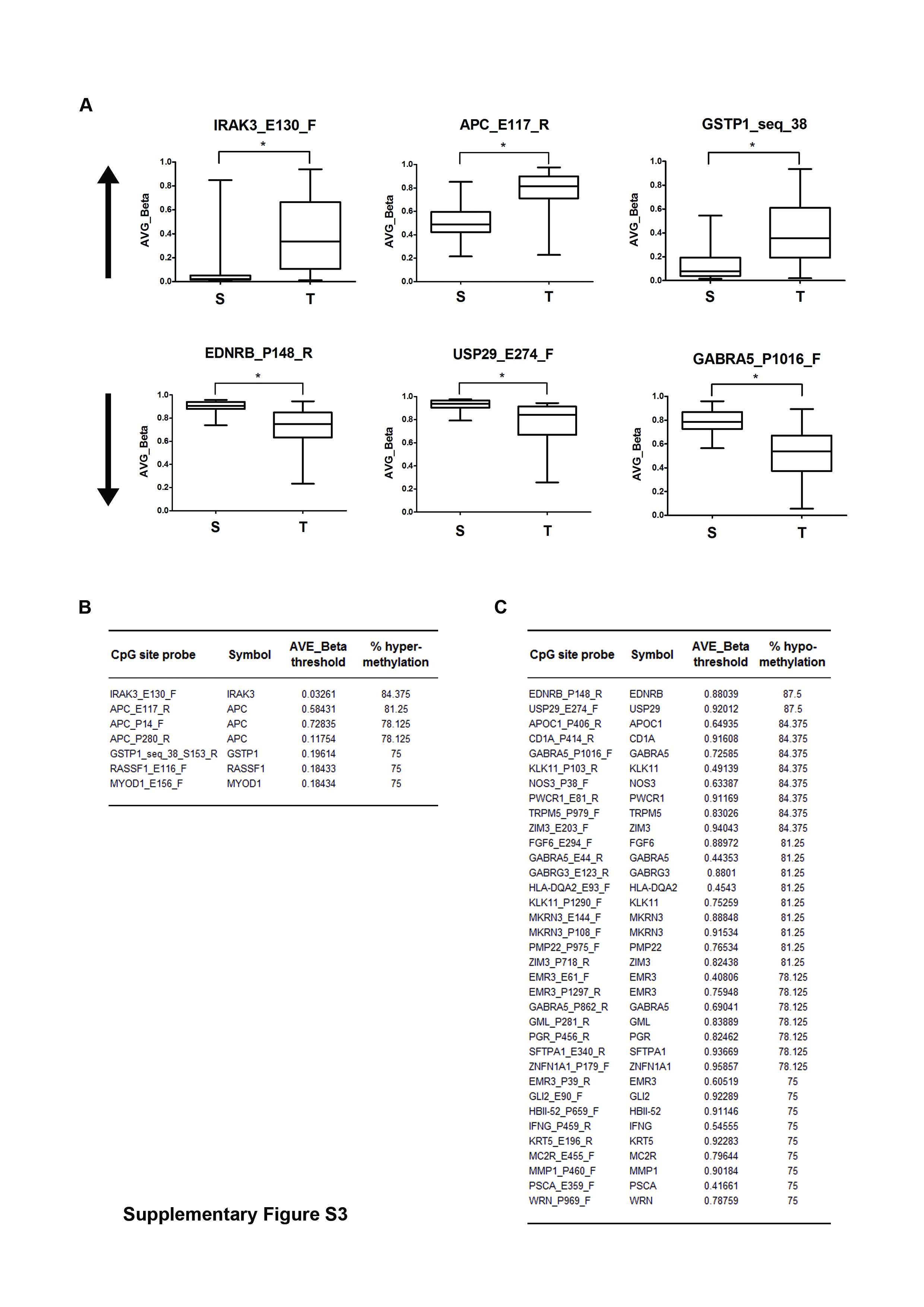

Supplement: Figure S3 — Analysis of frequency of methylation. AVG-Beta values in the surrounding tissues were used to define the percentiles 25 and 75 for each CpG site (see Methods). These percentiles were used as a reference to define the frequency of methylation in tumors. A. Box plots representing the 3 CpG sites with highest frequency of methylation in tumors (upper panel) and highest frequency of unmethylation in tumors (lower panel) calculated in this way. S = surrounding, T = tumor. (*) P value < 0.001. B. Table showing the CpG sites frequently methylated in more than 75% of the tumors relative to surrounding tissues. C. Table showing the CpG sites frequently unmethylated in more than 75% of the tumors relative to surrounding tissues. (2.56 MB TIF) [file pone.0009749.s003.tif]

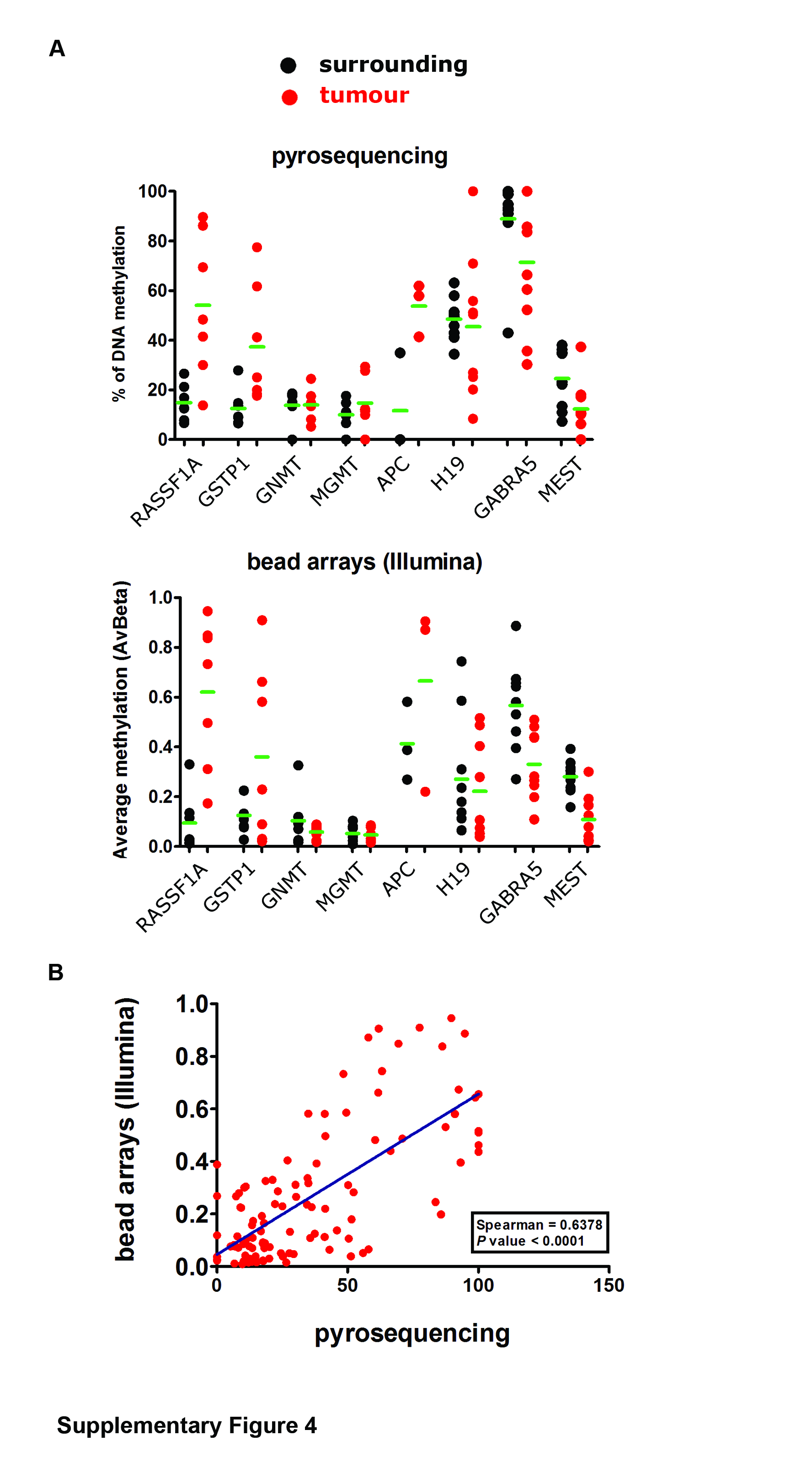

Supplement: Figure S4 — Validation of bead arrays by pyrosequencing A. Pyrosequencing assays were designed for the validation of 8 gene promoters differentially methylated between tumor and surrounding HCC samples (upper dot plot). The level of methylation is shown in a percentage scale. Primers were designed as described in Materials and Methods. A dot plot representing the corresponding levels of methylation (in a 0 to 1 scale) for the same genes in the bead arrays assay is shown in the lower panel. B. Correlation analysis from the data presented in (A). (1.45 MB TIF) [file pone.0009749.s004.tif]

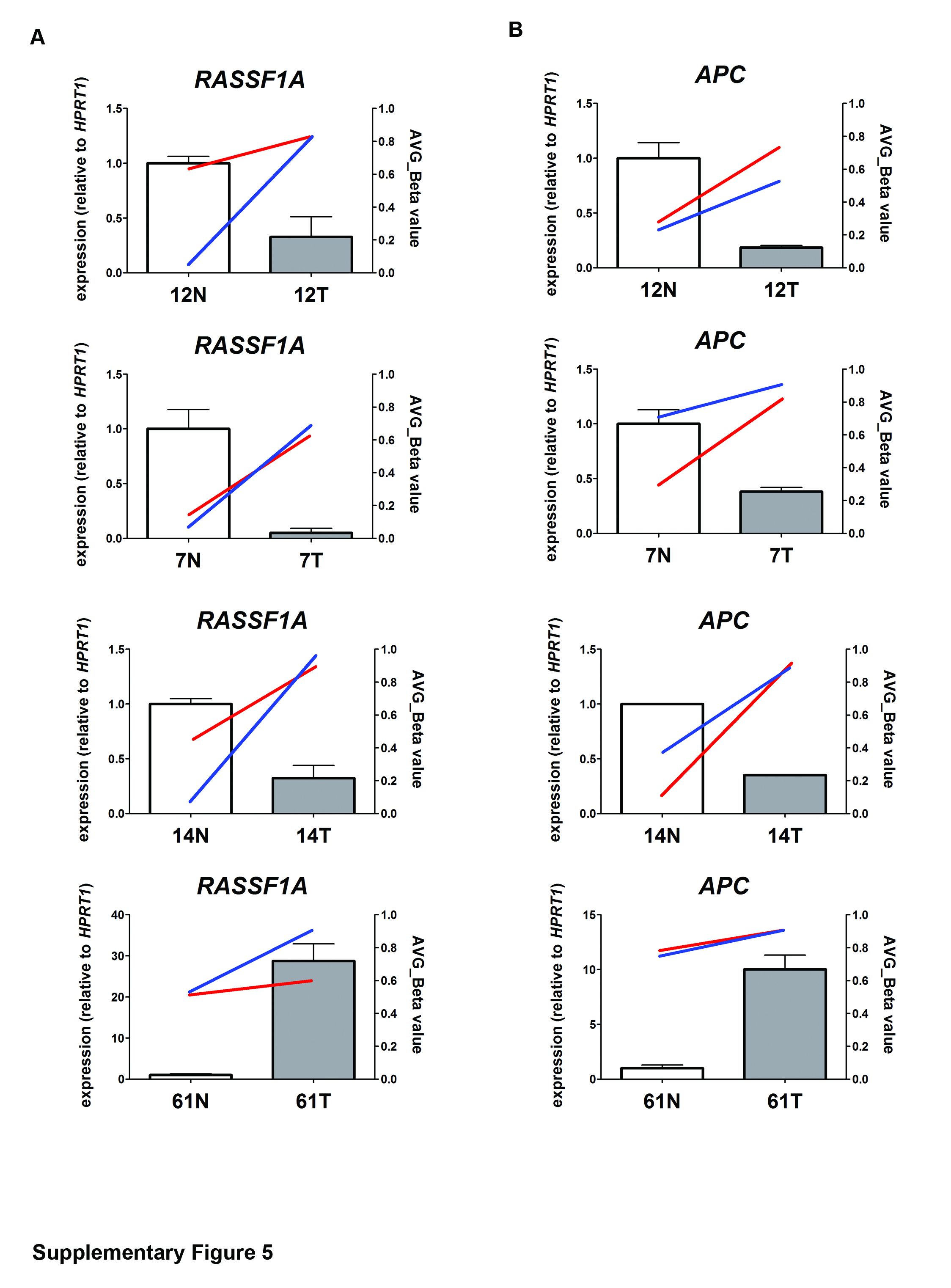

Supplement: Figure S5 — Validation of bead arrays by qRT-PCR Quantitative RT-PCR was performed for APC and RASSF1A in a subset of samples. The bars show a lower expression in the tumors relative to surrounding tissue in 3 out of 4 samples analyzed. In addition, inverse correlation with methylation is shown in each plot. Each line represents the AVG-Beta value obtained with bead arrays for 2 independent probes in the same promoter. Higher initial methylation is observed for the last sample, in which expression in tumors is higher than the matched surrounding tissue. (2.17 MB TIF) [file pone.0009749.s005.tif]
