# Supplementary material for: Hepatocellular Carcinoma Displays Distinct DNA Methylation Signatures with Potential as Clinical Predictors
Source: PLoS One. 2010 Mar 17;5(3):e9749. doi: 10.1371/journal.pone.0009749 (PMC2840036; doi:10.1371/journal.pone.0009749)
Supplement: Table S1 — Primers used for pyrosequencing. (0.06 MB DOC) [file pone.0009749.s006.doc]

## *Supplementary Table S1*. Primers used for pyrosequencing

|  |  |  |
| --- | --- | --- |
| **Name** | **Sequence (bisulfite-modified DNA)** | **Localization (UCSC)** |
|  |  |  |
| **GNMT (forward)** | 5’-GTATAAGGTATGGTTGTTTGG | chr6:43,036,749-43,036,791 |
| **GNMT (reverse)** | 5’-biotin-CTACTCCTAACCCTAATATCCC |
|  |  |  |
| **GSTP1 (forward)** | 5’-GTGATTTAGTATTGG | chr11:67,107,899-67,107,918 |
| **GSTP1 (reverse)** | 5’-biotin-AACTCTAAACCCCATC |
|  |  |  |
| **MGMT (forward)** | 5’-GTATTAGGAGGGGAGAGATT | chr10:131,154,992-131,155,016 |
| **MGMT (reverse)** | 5’-biotin-CCTTAATTTACCAAATAACCC |
|  |  |  |
| **RASSF1A (forward)** | 5’-AGTTTGGATTTTGGGGGAGG | chr3:50,353,262-50,353,297 |
| **RASSF1A (reverse)** | 5’-biotin-CAACTCAATAAACTCAAACTCCCC |
|  |  |  |
| **GABRA5 (forward)** | 5’- GTGAATGGTTAGAGTGAGAGAG | chr15:24,742,737-24,742,771 |
| **GABRA5 (reverse)** | 5’- biotin-CCCCAAAATATATATCCAAAAC |
|  |  |  |
| **H19 (forward)** | 5’- TTGAGGGGTAGAGGGAAGTGT | chr11:1,976,142-1,976,189 |
| **H19 (reverse)** | 5’- biotin-AATCTCCACTCCACTCCCAAC |
|  |  |  |
| **MEST (forward)** | 5’- GAAATTAGGGGAAGGGTTG | chr7:129,719,914-129,719,941 |
| **MEST (reverse)** | 5’- biotin-CCTTCTCCCTACCAAAC |
|  |  |  |
| **APC (forward)** | 5’-biotin-GAAATGGGGTAGGTGTTGG | chr5:112,101,578-112,101,612 |
| **APC (reverse)** | 5’-CTCCCCTTAACACTTCTACC |
|  |  |  |
