# Supplementary material for: Hepatocellular Carcinoma Displays Distinct DNA Methylation Signatures with Potential as Clinical Predictors
Source: PLoS One. 2010 Mar 17;5(3):e9749. doi: 10.1371/journal.pone.0009749 (PMC2840036; doi:10.1371/journal.pone.0009749)
Supplement: Table S2 — CpG sites differentially methylated in HCC tumor vs. surrounding tissue. (0.25 MB DOC) [file pone.0009749.s007.doc]

## *Supplementary Table S2. CpG sites differentially methylated in HCC tumor vs. surrounding tissue*

Significant CpG sites after paired class comparison analysis are shown (*P* < 0.001) in order of significance. Imprinted genes and their corresponding CpG probes are underlined. FDR= false discovery rate. Geometric mean (S/T) represents the level of methylation in surrounding/tumor tissue.

|  |  |  |  |  |  |
| --- | --- | --- | --- | --- | --- |
| **Probe ID** | **p-value** | **FDR** | **Geometric mean (S /T )** | **Symbol** | **Description** |
|  |  |  |  |  |  |
| 840 | < 1e-07 | < 1e-07 | 1.251 | [GABRA5](http://www.ncbi.nlm.nih.gov/entrez/query.fcgi?cmd=search&db=gene&term=APC) | Gamma-aminobutyric acid (GABA) A receptor, alpha 5 |
| 4022 | < 1e-07 | < 1e-07 | 0.845 | [APC](http://www.ncbi.nlm.nih.gov/entrez/query.fcgi?cmd=search&db=gene&term=ZIM3) | Adenomatosis polyposis coli |
| 69 | 0.0000 | 0.0000 | 1.140 | [APOC1](http://www.ncbi.nlm.nih.gov/entrez/query.fcgi?cmd=search&db=gene&term=RASSF1) | Apolipoprotein C-I |
| 3630 | 0.0000 | 0.0000 | 1.254 | [MC2R](http://www.ncbi.nlm.nih.gov/entrez/query.fcgi?cmd=search&db=gene&term=APOC1) | Melanocortin 2 receptor (adrenocorticotropic hormone) |
| 3865 | 0.0000 | 0.0000 | 0.724 | [RASSF1](http://www.ncbi.nlm.nih.gov/entrez/query.fcgi?cmd=search&db=gene&term=GABRA5) | Ras association (RalGDS/AF-6) domain family 1 |
| 5741 | 0.0000 | 0.0000 | 1.265 | [GML](http://www.ncbi.nlm.nih.gov/entrez/query.fcgi?cmd=search&db=gene&term=GML) | GPI anchored molecule like protein |
| 2205 | 0.0000 | 0.0001 | 1.210 | [ZIM3](http://www.ncbi.nlm.nih.gov/entrez/query.fcgi?cmd=search&db=gene&term=MC2R) | Zinc finger, imprinted 3 |
| 3295 | 0.0000 | 0.0001 | 1.199 | [GABRA5](http://www.ncbi.nlm.nih.gov/entrez/query.fcgi?cmd=search&db=gene&term=APC) | Gamma-aminobutyric acid (GABA) A receptor, alpha 5 |
| 3676 | 0.0000 | 0.0001 | 1.194 | [MKRN3](http://www.ncbi.nlm.nih.gov/entrez/query.fcgi?cmd=search&db=gene&term=MKRN3) | Makorin, ring finger protein, 3 |
| 1439 | 0.0000 | 0.0002 | 1.163 | [MKRN3](http://www.ncbi.nlm.nih.gov/entrez/query.fcgi?cmd=search&db=gene&term=MKRN3) | Makorin, ring finger protein, 3 |
| 4004 | 0.0000 | 0.0002 | 1.109 | [USP29](http://www.ncbi.nlm.nih.gov/entrez/query.fcgi?cmd=search&db=gene&term=USP29) | Ubiquitin specific peptidase 29 |
| 4208 | 0.0000 | 0.0004 | 1.210 | [NOTCH4](http://www.ncbi.nlm.nih.gov/entrez/query.fcgi?cmd=search&db=gene&term=PRSS1) | Notch homolog 4 (Drosophila) |
| 3986 | 0.0000 | 0.0004 | 1.182 | [TRPM5](http://www.ncbi.nlm.nih.gov/entrez/query.fcgi?cmd=search&db=gene&term=HLA-DQA2) | Transient receptor potential cation channel, subfamily M, member 5 |
| 1392 | 0.0000 | 0.0004 | 1.108 | [MEST](http://www.ncbi.nlm.nih.gov/entrez/query.fcgi?cmd=search&db=gene&term=TRPM5) | Mesoderm specific transcript homolog (mouse) |
| 844 | 0.0000 | 0.0004 | 1.167 | [GABRA5](http://www.ncbi.nlm.nih.gov/entrez/query.fcgi?cmd=search&db=gene&term=EDNRB) | Gamma-aminobutyric acid (GABA) A receptor, alpha 5 |
| 2155 | 0.0000 | 0.0004 | 1.136 | [TRPM5](http://www.ncbi.nlm.nih.gov/entrez/query.fcgi?cmd=search&db=gene&term=NOTCH4) | Transient receptor potential cation channel, subfamily M, member 5 |
| 740 | 0.0000 | 0.0004 | 1.182 | [EMR3](http://www.ncbi.nlm.nih.gov/entrez/query.fcgi?cmd=search&db=gene&term=EMR3) | Egf-like module containing, mucin-like, hormone receptor-like 3 |
| 3303 | 0.0000 | 0.0004 | 1.201 | [GABRG3](http://www.ncbi.nlm.nih.gov/entrez/query.fcgi?cmd=search&db=gene&term=GABRA5) | Gamma-aminobutyric acid (GABA) A receptor, gamma 3 |
| 700 | 0.0000 | 0.0004 | 1.125 | [EDNRB](http://www.ncbi.nlm.nih.gov/entrez/query.fcgi?cmd=search&db=gene&term=GABRG3) | Endothelin receptor type B |
| 2257 | 0.0000 | 0.0004 | 0.820 | [APC](http://www.ncbi.nlm.nih.gov/entrez/query.fcgi?cmd=search&db=gene&term=TRPM5) | Adenomatosis polyposis coli |
| 3697 | 0.0000 | 0.0005 | 0.805 | [MYOD1](http://www.ncbi.nlm.nih.gov/entrez/query.fcgi?cmd=search&db=gene&term=ZIM3) | Myogenic differentiation 1 |
| 4010 | 0.0000 | 0.0005 | 1.141 | [ZIM3](http://www.ncbi.nlm.nih.gov/entrez/query.fcgi?cmd=search&db=gene&term=CD1A) | Zinc finger, imprinted 3 |
| 3913 | 0.0000 | 0.0006 | 1.075 | [SFTPA1](http://www.ncbi.nlm.nih.gov/entrez/query.fcgi?cmd=search&db=gene&term=MSH3) | Surfactant protein A1 |
| 3222 | 0.0000 | 0.0007 | 1.171 | [EMR3](http://www.ncbi.nlm.nih.gov/entrez/query.fcgi?cmd=search&db=gene&term=FZD7) | Egf-like module containing, mucin-like, hormone receptor-like 3 |
| 3156 | 0.0000 | 0.0008 | 0.824 | [BMP4](http://www.ncbi.nlm.nih.gov/entrez/query.fcgi?cmd=search&db=gene&term=SFTPA1) | Bone morphogenetic protein 4 |
| 5698 | 0.0000 | 0.0008 | 1.178 | [PSCA](http://www.ncbi.nlm.nih.gov/entrez/query.fcgi?cmd=search&db=gene&term=HLA-DQA2) | Prostate stem cell antigen |
| 1600 | 0.0000 | 0.0009 | 0.838 | [FZD7](http://www.ncbi.nlm.nih.gov/entrez/query.fcgi?cmd=search&db=gene&term=KLK11) | Frizzled homolog 7 (Drosophila) |
| 3450 | 0.0000 | 0.0009 | 1.197 | [HLA-DQA2](http://www.ncbi.nlm.nih.gov/entrez/query.fcgi?cmd=search&db=gene&term=MYOD1) | Major histocompatibility complex, class II, DQ alpha 2 |
| 208 | 0.0000 | 0.0009 | 1.136 | [CD1A](http://www.ncbi.nlm.nih.gov/entrez/query.fcgi?cmd=search&db=gene&term=APC) | CD1a molecule |
| 4997 | 0.0000 | 0.0009 | 1.191 | [KLK11](http://www.ncbi.nlm.nih.gov/entrez/query.fcgi?cmd=search&db=gene&term=PWCR1) | Kallikrein-related peptidase 11 |
| 732 | 0.0000 | 0.0009 | 1.161 | [EMR3](http://www.ncbi.nlm.nih.gov/entrez/query.fcgi?cmd=search&db=gene&term=HBII-52) | Egf-like module containing, mucin-like, hormone receptor-like 3 |
| 4137 | 0.0000 | 0.0009 | 1.130 | [MSH3](http://www.ncbi.nlm.nih.gov/entrez/query.fcgi?cmd=search&db=gene&term=GABRA5) | MutS homolog 3 (E. coli) |
| 5003 | 0.0000 | 0.0010 | 1.148 | [KLK11](http://www.ncbi.nlm.nih.gov/entrez/query.fcgi?cmd=search&db=gene&term=KLK11) | Kallikrein-related peptidase 11 |
| 6140 | 0.0000 | 0.0011 | 0.812 | [CDKN2A](http://www.ncbi.nlm.nih.gov/entrez/query.fcgi?cmd=search&db=gene&term=MAGEA1) | Cyclin-dependent kinase inhibitor 2A (melanoma, p16, inhibits CDK4) |
| 649 | 0.0000 | 0.0012 | 0.836 | [IGF1R](http://www.ncbi.nlm.nih.gov/entrez/query.fcgi?cmd=search&db=gene&term=GFI1) | Insulin-like growth factor 1 receptor |
| 206 | 0.0000 | 0.0012 | 1.180 | [CD1A](http://www.ncbi.nlm.nih.gov/entrez/query.fcgi?cmd=search&db=gene&term=NOS3) | CD1a molecule |
| 4201 | 0.0000 | 0.0013 | 1.136 | [NOS3](http://www.ncbi.nlm.nih.gov/entrez/query.fcgi?cmd=search&db=gene&term=EMR3) | Nitric oxide synthase 3 (endothelial cell) |
| 5790 | 0.0000 | 0.0015 | 1.152 | [IFNG](http://www.ncbi.nlm.nih.gov/entrez/query.fcgi?cmd=search&db=gene&term=PGR) | Interferon, gamma |
| 2259 | 0.0000 | 0.0015 | 0.852 | [APC](http://www.ncbi.nlm.nih.gov/entrez/query.fcgi?cmd=search&db=gene&term=MMP9) | Adenomatosis polyposis coli |
| 4304 | 0.0000 | 0.0015 | 1.086 | [MMP1](http://www.ncbi.nlm.nih.gov/entrez/query.fcgi?cmd=search&db=gene&term=BMP4) | Matrix metallopeptidase 1 (interstitial collagenase) |
| 5021 | 0.0001 | 0.0018 | 1.104 | [SPI1](http://www.ncbi.nlm.nih.gov/entrez/query.fcgi?cmd=search&db=gene&term=EMR3) | Spleen focus forming virus (SFFV) proviral integration oncogene spi1 |
| 3846 | 0.0001 | 0.0018 | 1.066 | [PWCR1](http://www.ncbi.nlm.nih.gov/entrez/query.fcgi?cmd=search&db=gene&term=MEST) | Prader-Willi syndrome chromosome region 1 |
| 4154 | 0.0001 | 0.0020 | 1.157 | [PGR](http://www.ncbi.nlm.nih.gov/entrez/query.fcgi?cmd=search&db=gene&term=NQO1) | Progesterone receptor |
| 1650 | 0.0001 | 0.0021 | 1.146 | [WNT8B](http://www.ncbi.nlm.nih.gov/entrez/query.fcgi?cmd=search&db=gene&term=TRPM5) | Wingless-type MMTV integration site family, member 8B |
| 609 | 0.0001 | 0.0021 | 1.111 | [GLI2](http://www.ncbi.nlm.nih.gov/entrez/query.fcgi?cmd=search&db=gene&term=CTAG1B) | GLI-Kruppel family member GLI2 |
| 460 | 0.0001 | 0.0021 | 1.133 | [FGF6](http://www.ncbi.nlm.nih.gov/entrez/query.fcgi?cmd=search&db=gene&term=PSCA) | Fibroblast growth factor 6 |
| 3879 | 0.0001 | 0.0021 | 1.111 | [RUNX3](http://www.ncbi.nlm.nih.gov/entrez/query.fcgi?cmd=search&db=gene&term=MMP1) | Runt-related transcription factor 3 |
| 5510 | 0.0001 | 0.0021 | 1.114 | [CYP2E1](http://www.ncbi.nlm.nih.gov/entrez/query.fcgi?cmd=search&db=gene&term=FGF6) | Cytochrome P450, family 2, subfamily E, polypeptide 1 |
| 3375 | 0.0001 | 0.0025 | 1.131 | [HBII-52](http://www.ncbi.nlm.nih.gov/entrez/query.fcgi?cmd=search&db=gene&term=HBII-52) | Small nucleolar RNA, C/D box 115 cluster |
| 4300 | 0.0001 | 0.0025 | 1.147 | [CYP2E1](http://www.ncbi.nlm.nih.gov/entrez/query.fcgi?cmd=search&db=gene&term=GLI2) | Cytochrome P450, family 2, subfamily E, polypeptide 1 |
| 171 | 0.0001 | 0.0025 | 1.118 | [C4B](http://www.ncbi.nlm.nih.gov/entrez/query.fcgi?cmd=search&db=gene&term=CDKN2A) | Complement component 4B (Chido blood group) |
| 5877 | 0.0001 | 0.0025 | 1.114 | [GFAP](http://www.ncbi.nlm.nih.gov/entrez/query.fcgi?cmd=search&db=gene&term=KRT5) | Glial fibrillary acidic protein |
| 963 | 0.0001 | 0.0025 | 1.102 | [HBII-52](http://www.ncbi.nlm.nih.gov/entrez/query.fcgi?cmd=search&db=gene&term=PADI4) | Small nucleolar RNA, C/D box 115 cluster |
| 1655 | 0.0001 | 0.0025 | 1.124 | [PADI4](http://www.ncbi.nlm.nih.gov/entrez/query.fcgi?cmd=search&db=gene&term=CD1A) | Peptidyl arginine deiminase, type IV |
| 2813 | 0.0001 | 0.0025 | 1.163 | [PGR](http://www.ncbi.nlm.nih.gov/entrez/query.fcgi?cmd=search&db=gene&term=CYP2E1) | Progesterone receptor |
| 5551 | 0.0001 | 0.0027 | 0.845 | [FZD9](http://www.ncbi.nlm.nih.gov/entrez/query.fcgi?cmd=search&db=gene&term=PMP22) | Frizzled homolog 9 (Drosophila) |
| 1653 | 0.0001 | 0.0028 | 1.144 | [ZNFN1A1](http://www.ncbi.nlm.nih.gov/entrez/query.fcgi?cmd=search&db=gene&term=IRAK3) | IKAROS family zinc finger 1 (Ikaros) |
| 4012 | 0.0001 | 0.0028 | 0.849 | [ZMYND10](http://www.ncbi.nlm.nih.gov/entrez/query.fcgi?cmd=search&db=gene&term=KRT1) | Zinc finger, MYND-type containing 10 |
| 75 | 0.0001 | 0.0029 | 0.844 | [ALOX12](http://www.ncbi.nlm.nih.gov/entrez/query.fcgi?cmd=search&db=gene&term=SPI1) | Arachidonate 12-lipoxygenase |
| 5013 | 0.0001 | 0.0029 | 1.123 | [KRT1](http://www.ncbi.nlm.nih.gov/entrez/query.fcgi?cmd=search&db=gene&term=RUNX3) | Keratin 1 (epidermolytic hyperkeratosis) |
| 2797 | 0.0001 | 0.0029 | 1.123 | [KIAA0125](http://www.ncbi.nlm.nih.gov/entrez/query.fcgi?cmd=search&db=gene&term=MAGEC3) | KIAA0125 |
| 5591 | 0.0002 | 0.0030 | 0.820 | [IRAK3](http://www.ncbi.nlm.nih.gov/entrez/query.fcgi?cmd=search&db=gene&term=ZMYND10) | Interleukin-1 receptor-associated kinase 3 |
| 1581 | 0.0002 | 0.0031 | 1.149 | [NBL1](http://www.ncbi.nlm.nih.gov/entrez/query.fcgi?cmd=search&db=gene&term=IGF1R) | Neuroblastoma, suppression of tumorigenicity 1 |
| 3245 | 0.0002 | 0.0031 | 0.819 | [COL18A1](http://www.ncbi.nlm.nih.gov/entrez/query.fcgi?cmd=search&db=gene&term=PGR) | Collagen, type XVIII, alpha 1 |
| 2976 | 0.0002 | 0.0032 | 1.143 | [AATK](http://www.ncbi.nlm.nih.gov/entrez/query.fcgi?cmd=search&db=gene&term=ALOX12) | Apoptosis-associated tyrosine kinase |
| 1718 | 0.0002 | 0.0033 | 1.143 | [PMP22](http://www.ncbi.nlm.nih.gov/entrez/query.fcgi?cmd=search&db=gene&term=FYN) | Peripheral myelin protein 22 |
| 5476 | 0.0002 | 0.0034 | 1.140 | [CDH17](http://www.ncbi.nlm.nih.gov/entrez/query.fcgi?cmd=search&db=gene&term=ZNFN1A1) | Cadherin 17, LI cadherin (liver-intestine) |
| 3001 | 0.0002 | 0.0035 | 0.838 | [ALOX12](http://www.ncbi.nlm.nih.gov/entrez/query.fcgi?cmd=search&db=gene&term=GSTM2) | Arachidonate 12-lipoxygenase |
| 2803 | 0.0002 | 0.0036 | 1.118 | [KRT5](http://www.ncbi.nlm.nih.gov/entrez/query.fcgi?cmd=search&db=gene&term=HLA-DOA) | Keratin 5 (epidermolysis bullosa simplex, Dowling-Meara/Kobner/Weber-Cockayne types) |
| 935 | 0.0002 | 0.0036 | 1.116 | [MMP9](http://www.ncbi.nlm.nih.gov/entrez/query.fcgi?cmd=search&db=gene&term=C4B) | Matrix metallopeptidase 9 (gelatinase B, 92kDa gelatinase, 92kDa type IV collagenase) |
| 3592 | 0.0002 | 0.0036 | 1.162 | [DCN](http://www.ncbi.nlm.nih.gov/entrez/query.fcgi?cmd=search&db=gene&term=EGF) | Decorin |
| 4982 | 0.0002 | 0.0037 | 0.868 | [SH3BP2](http://www.ncbi.nlm.nih.gov/entrez/query.fcgi?cmd=search&db=gene&term=GFAP) | SH3-domain binding protein 2 |
| 3060 | 0.0002 | 0.0037 | 1.131 | [PRSS1](http://www.ncbi.nlm.nih.gov/entrez/query.fcgi?cmd=search&db=gene&term=KIAA0125) | Protease, serine, 1 (trypsin 1) |
| 946 | 0.0002 | 0.0038 | 0.842 | [GSTM2](http://www.ncbi.nlm.nih.gov/entrez/query.fcgi?cmd=search&db=gene&term=RUNX3) | Glutathione S-transferase M2 (muscle) |
| 3354 | 0.0002 | 0.0040 | 0.845 | [GSTM2](http://www.ncbi.nlm.nih.gov/entrez/query.fcgi?cmd=search&db=gene&term=NGFR) | Glutathione S-transferase M2 (muscle) |
| 2811 | 0.0002 | 0.0040 | 1.124 | [PGR](http://www.ncbi.nlm.nih.gov/entrez/query.fcgi?cmd=search&db=gene&term=CDH17) | Progesterone receptor |
| 4312 | 0.0003 | 0.0046 | 1.109 | [DES](http://www.ncbi.nlm.nih.gov/entrez/query.fcgi?cmd=search&db=gene&term=WNT8B) | Desmin |
| 4197 | 0.0003 | 0.0047 | 0.887 | [NGFR](http://www.ncbi.nlm.nih.gov/entrez/query.fcgi?cmd=search&db=gene&term=MEST) | Nerve growth factor receptor (TNFR superfamily, member 16) |
| 4002 | 0.0003 | 0.0052 | 0.850 | [FES](http://www.ncbi.nlm.nih.gov/entrez/query.fcgi?cmd=search&db=gene&term=PLG) | Feline sarcoma oncogene |
| 1869 | 0.0003 | 0.0052 | 1.127 | [RUNX3](http://www.ncbi.nlm.nih.gov/entrez/query.fcgi?cmd=search&db=gene&term=FZD9) | Runt-related transcription factor 3 |
| 4161 | 0.0004 | 0.0053 | 0.828 | [MST1R](http://www.ncbi.nlm.nih.gov/entrez/query.fcgi?cmd=search&db=gene&term=CYP2E1) | Macrophage stimulating 1 receptor (c-met-related tyrosine kinase) |
| 1006 | 0.0004 | 0.0053 | 1.115 | [HLA-DOA](http://www.ncbi.nlm.nih.gov/entrez/query.fcgi?cmd=search&db=gene&term=EFNB3) | Major histocompatibility complex, class II, DO alpha |
| 2939 | 0.0004 | 0.0053 | 1.090 | [EPHX1](http://www.ncbi.nlm.nih.gov/entrez/query.fcgi?cmd=search&db=gene&term=STK23) | Epoxide hydrolase 1, microsomal (xenobiotic) |
| 4279 | 0.0004 | 0.0056 | 1.153 | [CDH17](http://www.ncbi.nlm.nih.gov/entrez/query.fcgi?cmd=search&db=gene&term=PGR) | Cadherin 17, LI cadherin (liver-intestine) |
| 3050 | 0.0004 | 0.0059 | 0.903 | [BMP4](http://www.ncbi.nlm.nih.gov/entrez/query.fcgi?cmd=search&db=gene&term=NOTCH3) | Bone morphogenetic protein 4 |
| 4268 | 0.0004 | 0.0059 | 1.114 | [PLG](http://www.ncbi.nlm.nih.gov/entrez/query.fcgi?cmd=search&db=gene&term=PLA2G2A) | Plasminogen |
| 3814 | 0.0004 | 0.0059 | 1.145 | [EGF](http://www.ncbi.nlm.nih.gov/entrez/query.fcgi?cmd=search&db=gene&term=CDH17) | Epidermal growth factor (beta-urogastrone) |
| 2794 | 0.0004 | 0.0059 | 1.150 | ITK | IL2-inducible T-cell kinase |
| 3497 | 0.0004 | 0.0060 | 1.067 | EGF | Epidermal growth factor (beta-urogastrone) |
| 4976 | 0.0005 | 0.0061 | 1.125 | PLA2G2A | Phospholipase A2, group IIA (platelets, synovial fluid) |
| 5257 | 0.0005 | 0.0063 | 1.084 | TFF2 | Trefoil factor 2 (spasmolytic protein 1) |
| 2137 | 0.0005 | 0.0064 | 0.881 | TMEFF2 | Transmembrane protein with EGF-like and two follistatin-like domains 2 |
| 2390 | 0.0005 | 0.0066 | 1.119 | MUSK | Muscle, skeletal, receptor tyrosine kinase |
| 3804 | 0.0005 | 0.0067 | 0.871 | FLT4 | Fms-related tyrosine kinase 4 |
| 1790 | 0.0006 | 0.0072 | 1.097 | PWCR1 | Prader-Willi syndrome chromosome region 1 |
| 6078 | 0.0006 | 0.0075 | 1.050 | ZNFN1A1 | IKAROS family zinc finger 1 (Ikaros) |
| 4850 | 0.0006 | 0.0075 | 1.142 | MMP3 | Matrix metallopeptidase 3 (stromelysin 1, progelatinase) |
| 2619 | 0.0007 | 0.0083 | 0.843 | GSTP1 | Glutathione S-transferase pi |
| 1772 | 0.0007 | 0.0083 | 1.110 | PTHR1 | Parathyroid hormone receptor 1 |
| 2885 | 0.0007 | 0.0088 | 0.875 | TERT | Telomerase reverse transcriptase |
| 5078 | 0.0007 | 0.0088 | 1.141 | NID1 | Nidogen 1 |
| 377 | 0.0007 | 0.0088 | 1.095 | CPA4 | Carboxypeptidase A4 |
| 2204 | 0.0007 | 0.0088 | 1.043 | ZIM3 | Zinc finger, imprinted 3 |
| 300 | 0.0007 | 0.0088 | 1.143 | CHI3L2 | Chitinase 3-like 2 |
| 605 | 0.0008 | 0.0088 | 0.865 | FES | Feline sarcoma oncogene |
| 1730 | 0.0008 | 0.0088 | 1.155 | PMP22 | Peripheral myelin protein 22 |
| 4107 | 0.0008 | 0.0088 | 0.860 | HOXA9 | Homeobox A9 |
| 4205 | 0.0008 | 0.0088 | 1.138 | IL2 | Interleukin 2 |
| 4018 | 0.0008 | 0.0088 | 0.881 | ZP3 | Zona pellucida glycoprotein 3 (sperm receptor) |
| 1595 | 0.0008 | 0.0089 | 1.119 | NDN | Necdin homolog (mouse) |
| 3778 | 0.0008 | 0.0089 | 1.147 | PI3 | Peptidase inhibitor 3, skin-derived (SKALP) |
| 6038 | 0.0008 | 0.0089 | 0.853 | GSTP1 | Glutathione S-transferase pi |
| 2291 | 0.0008 | 0.0089 | 1.138 | ITK | IL2-inducible T-cell kinase |
| 594 | 0.0008 | 0.0089 | 1.062 | CTLA4 | Cytotoxic T-lymphocyte-associated protein 4 |
| 715 | 0.0008 | 0.0089 | 1.075 | EDNRB | Endothelin receptor type B |
| 2190 | 0.0009 | 0.0092 | 1.122 | VAMP8 | Vesicle-associated membrane protein 8 (endobrevin) |
| 2129 | 0.0009 | 0.0092 | 0.888 | TMEFF1 | Transmembrane protein with EGF-like and two follistatin-like domains 1 |
| 1629 | 0.0009 | 0.0092 | 1.129 | IFNG | Interferon, gamma |
| 2329 | 0.0009 | 0.0092 | 0.896 | CCND2 | Cyclin D2 |
| 764 | 0.0009 | 0.0092 | 0.901 | F2R | Coagulation factor II (thrombin) receptor |
| 1390 | 0.0009 | 0.0092 | 1.100 | MEST | Mesoderm specific transcript homolog (mouse) |
| 1203 | 0.0009 | 0.0093 | 1.126 | INS | Insulin |
| 4874 | 0.0010 | 0.0096 | 1.098 | FASTK | Fas-activated serine/threonine kinase |
| 623 | 0.0010 | 0.0096 | 0.841 | FRZB | Frizzled-related protein |
|  |  |  |  |  |  |
