# Supplementary material for: Hepatocellular Carcinoma Displays Distinct DNA Methylation Signatures with Potential as Clinical Predictors
Source: PLoS One. 2010 Mar 17;5(3):e9749. doi: 10.1371/journal.pone.0009749 (PMC2840036; doi:10.1371/journal.pone.0009749)
Supplement: Table S3 — CpG sites differentially methylated in HCC according to risk factor exposure. (0.07 MB DOC) [file pone.0009749.s008.doc]

## *Supplementary Table S3. CpG sites differentially methylated in HCC according to risk factor exposure*

Significant CpG sites after paired class comparison analysis are shown (*P* < 0.01) in order of significance. Geometric mean representing the level of methylation in each category is shown (HBV or HCV infection, EtOH = ethanol consumption, and Unknown = unknown risk factor).

|  |  |  |  |  |  |  |  |
| --- | --- | --- | --- | --- | --- | --- | --- |
|  |  | **HCC Risk Factor** | | | |  |  |
| **Probe** | **Symbol** | **HBV** | **HCV** | **EtOH** | **Unknown** | ***P*-value** | **Description** |
|  |  |  |  |  |  |  |  |
| 2425 | [**DCC**](http://www.ncbi.nlm.nih.gov/entrez/query.fcgi?cmd=search&db=gene&term=DCC) | 1.03 | 1.25 | 1.05 | 1.03 | 0.0004 | Deleted in colorectal carcinoma |
| 2003 | [**SLC5A8**](http://www.ncbi.nlm.nih.gov/entrez/query.fcgi?cmd=search&db=gene&term=NAT2) | 1.05 | 1.18 | 1.07 | 1.02 | 0.0010 | Solute carrier family 5 (iodide transporter), member 8 |
| 1826 | [**RASGRF1**](http://www.ncbi.nlm.nih.gov/entrez/query.fcgi?cmd=search&db=gene&term=RYK) | 1.03 | 1.10 | 1.03 | 1.02 | 0.0019 | Ras protein-specific guanine nucleotide-releasing factor 1 |
| 4177 | [**NAT2**](http://www.ncbi.nlm.nih.gov/entrez/query.fcgi?cmd=search&db=gene&term=RASGRF1) | 1.36 | 1.47 | 1.14 | 1.17 | 0.0021 | N-acetyltransferase 2 (arylamine N-acetyltransferase) |
| 5179 | [**RYK**](http://www.ncbi.nlm.nih.gov/entrez/query.fcgi?cmd=search&db=gene&term=SLC5A8) | 1.04 | 1.10 | 1.15 | 1.03 | 0.0026 | RYK receptor-like tyrosine kinase |
| 634 | [**DIO3**](http://www.ncbi.nlm.nih.gov/entrez/query.fcgi?cmd=search&db=gene&term=DIO3) | 1.51 | 1.17 | 1.30 | 1.35 | 0.0036 | Deiodinase, iodothyronine, type III |
| 1109 | [**TNFSF10**](http://www.ncbi.nlm.nih.gov/entrez/query.fcgi?cmd=search&db=gene&term=TNFRSF10C) | 1.04 | 1.13 | 1.03 | 1.03 | 0.0056 | Tumor necrosis factor (ligand) superfamily, member 10 |
| 2144 | [**TNFRSF10C**](http://www.ncbi.nlm.nih.gov/entrez/query.fcgi?cmd=search&db=gene&term=MLH3) | 1.82 | 1.55 | 1.84 | 1.88 | 0.0068 | Tumor necrosis factor receptor superfamily, member 10c, decoy without an intracellular domain |
| 2409 | [**CSPG2**](http://www.ncbi.nlm.nih.gov/entrez/query.fcgi?cmd=search&db=gene&term=TNFSF10) | 1.12 | 1.35 | 1.05 | 1.11 | 0.0072 | Chondroitin sulfate proteoglycan 2; versican |
| 285 | [**CHGA**](http://www.ncbi.nlm.nih.gov/entrez/query.fcgi?cmd=search&db=gene&term=STAT5A) | 1.08 | 1.13 | 1.16 | 1.04 | 0.0075 | Chromogranin A (parathyroid secretory protein 1) |
| 5243 | [**STAT5A**](http://www.ncbi.nlm.nih.gov/entrez/query.fcgi?cmd=search&db=gene&term=CSPG2) | 1.42 | 1.69 | 1.71 | 1.78 | 0.0084 | Signal transducer and activator of transcription 5A |
| 1543 | [**MLH3**](http://www.ncbi.nlm.nih.gov/entrez/query.fcgi?cmd=search&db=gene&term=CHGA) | 1.01 | 1.01 | 1.04 | 1.03 | 0.0096 | MutL homolog 3 (E. coli) |
| 4957 | [**NTRK3**](http://www.ncbi.nlm.nih.gov/entrez/query.fcgi?cmd=search&db=gene&term=FGFR2) | 1.09 | 1.28 | 1.20 | 1.02 | 0.0097 | Neurotrophic tyrosine kinase, receptor, type 3 |
|  |  |  |  |  |  |  |  |
